# Blount’s disease successfully treated with intraepiphyseal osteotomy with elevation of the medial plateau of the tibia—a case report with 65 years’ follow-up

**DOI:** 10.1080/17453674.2018.1516179

**Published:** 2018-10-17

**Authors:** Terje Terjesen, Darko Anticevic

**Affiliations:** 1 Orthopaedic Department, Oslo University Hospital, Rikshospitalet, Oslo, Norway;; 2 Department of Paediatric Orthopaedics, Zagreb Children’s Hospital, Zagreb, Croatia

A girl born in 1938 with Blount’s disease and pronounced varus deformity of her right knee underwent osteotomies of the proximal tibia and fibula with overcorrection into valgus at the age of 8 years. Because of relapse, a similar operation with overcorrection to 10° valgus was performed 4 years later. However, again she had relapse of her varus deformity (Figure 1).

**Figure 1 a.**
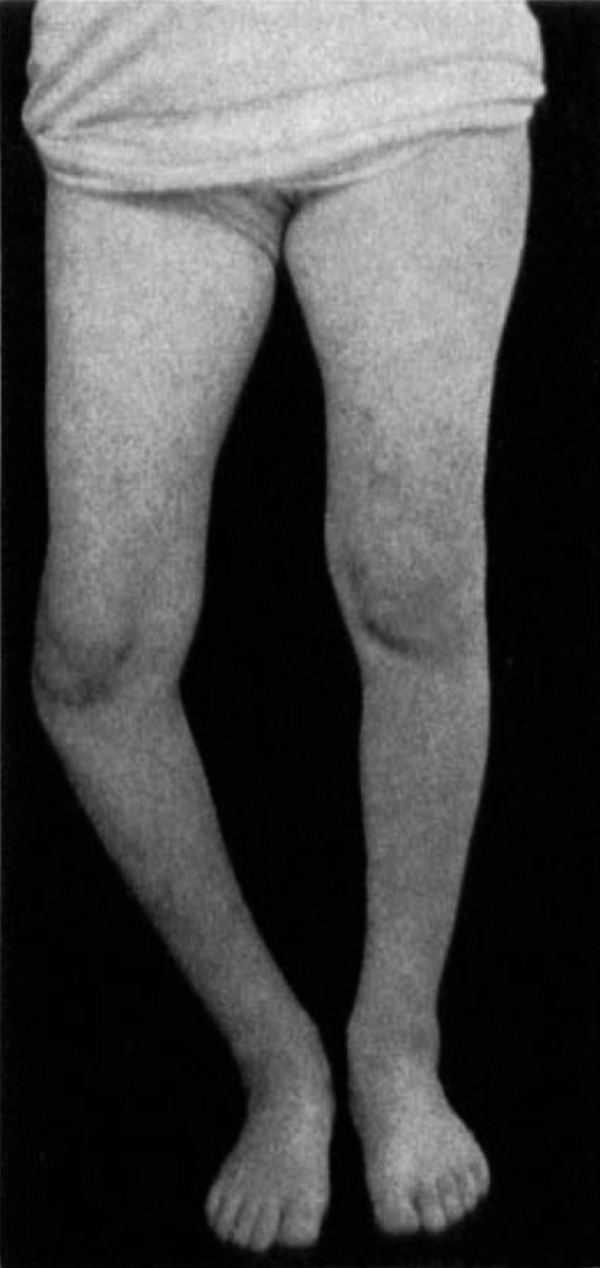
Preoperative picture of the patient at the age of 13 years, showing pronounced varus deformity of her right leg.

**Figure 1 b.**
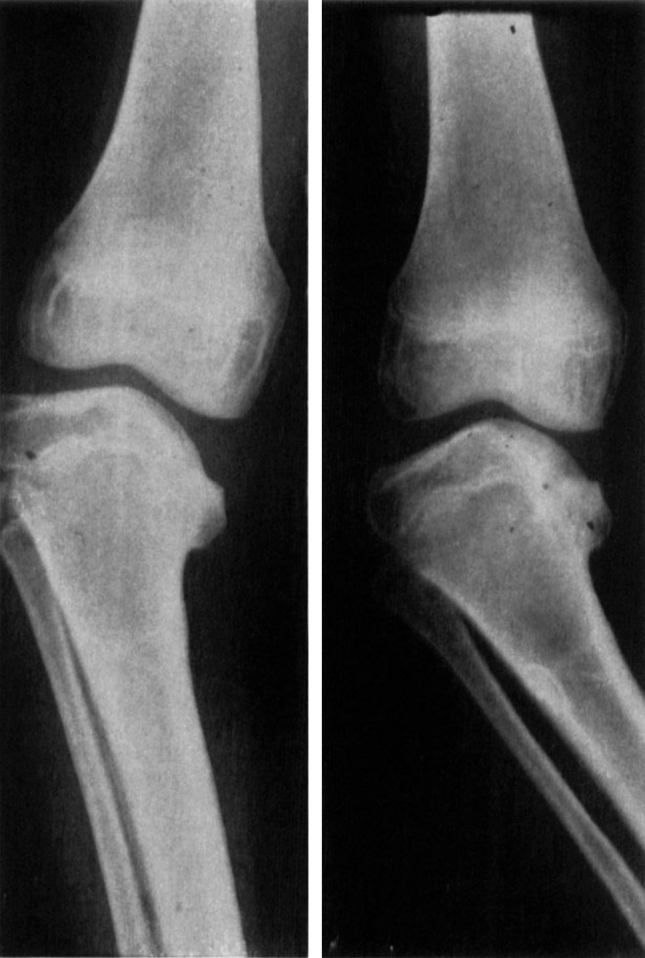
Preoperative radiographs, without weight-bearing (left). During weight-bearing (right) varus is increased because the defective medial condyle provides poor support for the femoral condyle. Reprinted from Støren (1969).

**Figure 1 c.**
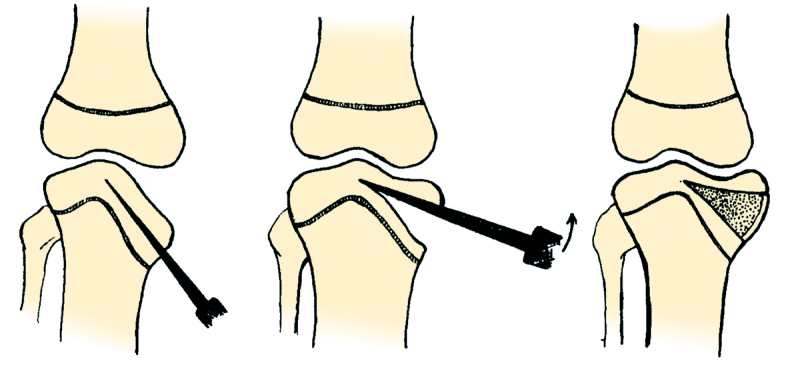
Operative technique. The chiseling is done proximal to the epiphyseal line and proceeds to the midline (left). The fragment is slowly elevated, lifting the whole fragment in one piece (middle). Solid bone grafts from the iliac crest, large enough to force the fragment into maximal elevation, are wedged under the fragment under maximal forced valgus of the knee (right).

**Figure 1 d.**
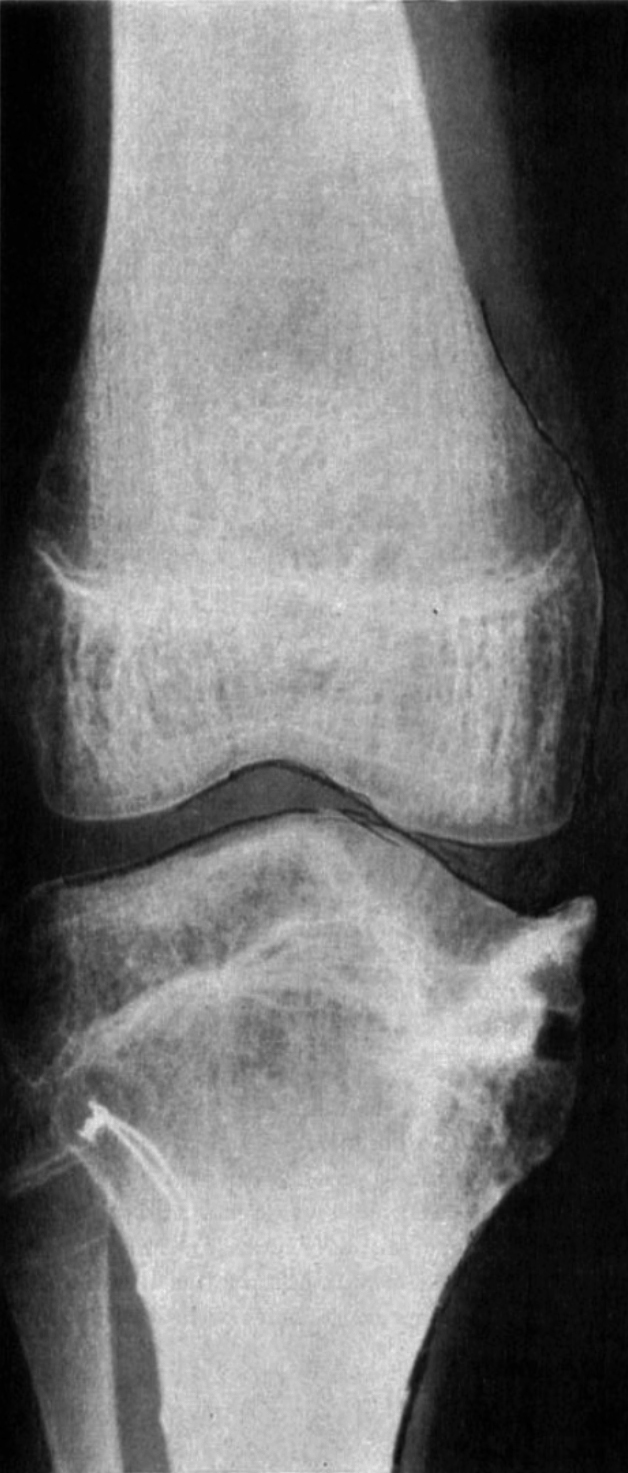
Radiograph 1 year after operative elevation of the medial tibial joint surface, showing consolidation and good correction of the varus deformity.

In 1951 (patient age 13 years) an intraepiphyseal osteotomy of the medial tibial condyle, with elevation of the medial tibial plateau, was performed. The surgical technique was precisely described by Støren (1969) (Figure 1). The medial tibial condyle was exposed through a medial longitudinal incision about 12 cm long. The joint was not opened, except for a minor slit for orientation. The chiseling was done parallel to the sloping epiphyseal line at safe proximal distance from the line. Care was also taken to avoid getting too close to the joint surface. Several thin-bladed chisels, 3 cm wide, were used simultaneously. The chiseling proceeded to the midline, under radiographic control. The medial epicondylar fragment was carefully lifted, the final elevation being carried out with a wide chisel covering the whole fragment. With the knee in full valgus, large bone transplants from the iliac crest were wedged under the fragment under maximal elevation. A plaster cast was applied under forced valgus and maintained for 12 weeks. Weight-bearing was avoided for 1 year. The osteotomy healed without any complications and gave good correction of the deformity (Figure 1). The treatment was published as a case report in Acta Orthopaedica Scandinavica (Støren 1969), with a follow-up time of 18 years. At that time the patient had good function of the knee and no pain.

We managed to locate the patient and in November 2016, with a follow-up of 65 years (patient age 78 years), she underwent a clinical and radiographic examination. She was satisfied with the outcome, had good gait function and no pain on her usual activities. She could do “everything” (skiing, bicycling, swimming), but felt tiredness in her leg and felt that the knee was a bit unstable when walking on rough ground. The mobility of the right knee was good (Figure 2), with –5° of extension and 130° of flexion (5° and 140°, respectively, in her left knee). The radiographs showed moderate osteoarthritis (OA) of the knee. Long-leg standing radiographs showed satisfactory position with 2° varus in her right knee and 0° in the left (Figure 2). There was a moderate leg length discrepancy (LLD) with the right leg 1.5 cm shorter.

**Figure 2 a.**
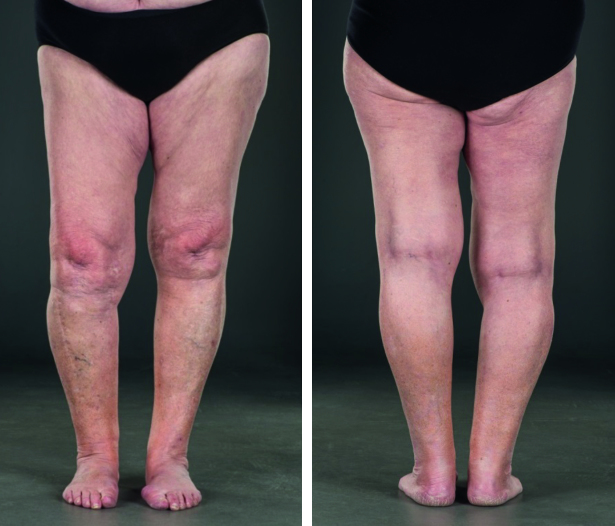
Pictures at 65 years of follow-up (patient age 78 years), showing slight varus deformity of the right leg.

**Figure 2 b.**
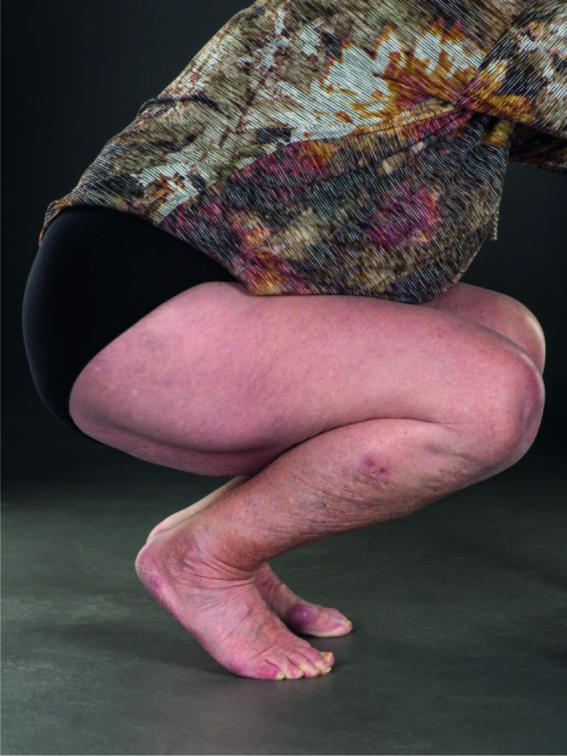
At 65 years follow-up the patient had almost full knee flexion.

**Figure 2 c.**
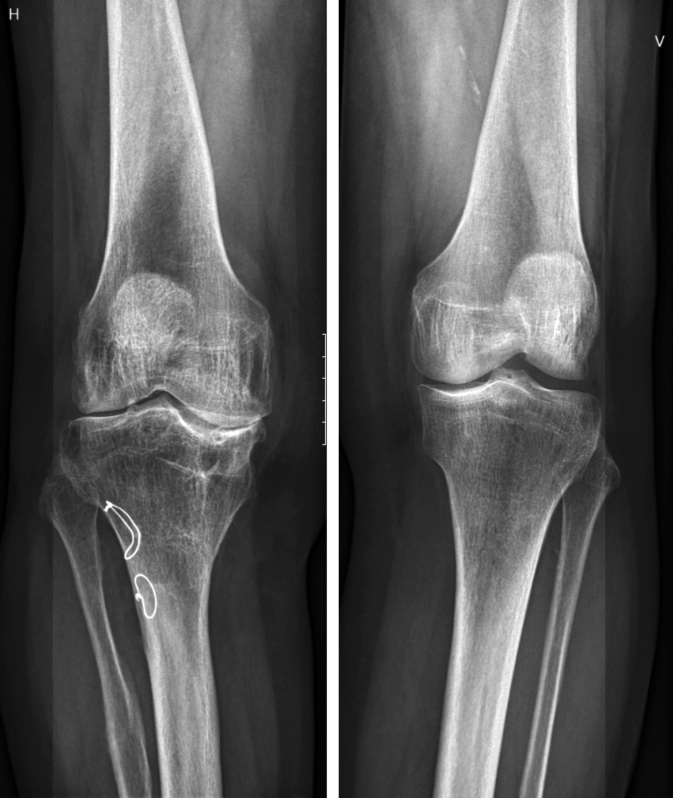
Radiographs at 65 years follow-up, showing moderate osteoarthritis of the right knee.

**Figure 2 d.**
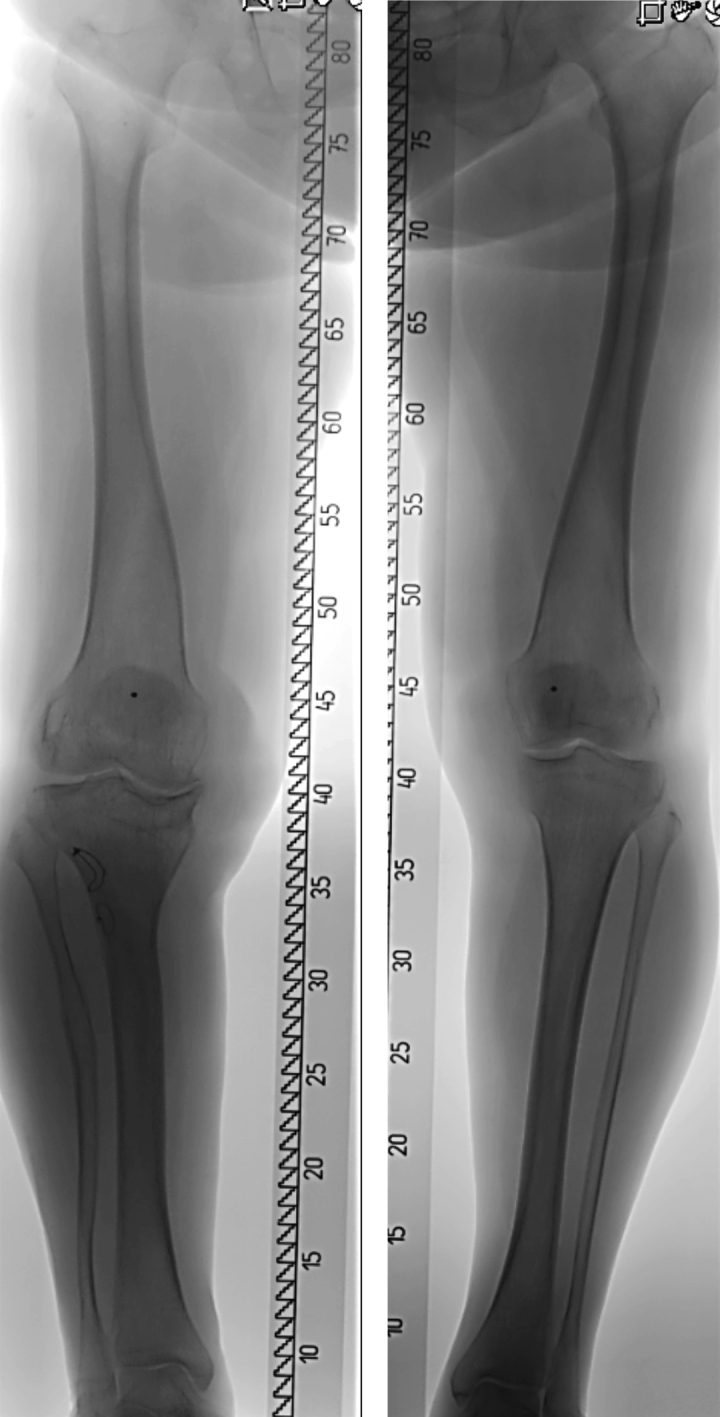
Long-leg standing radiographs at 65 years follow-up, showing slight varus of the right knee and leg length discrepancy of 1.5 cm with the right leg shorter.

## Discussion

Blount’s disease (tibia vara) is a disorder of growth that affects the medial aspect of the proximal end of the tibia leading to a progressive varus deformity. Langenskiöld and Riska (1964) maintained that, without operative treatment, the varus deformity usually progresses; thus early osteotomy is imperative. They recommended curved osteotomy of the proximal tibial metaphysis and osteotomy of the proximal fibula at the age of 2–8 years; this procedure cured the condition permanently in most patients. Recurrence usually occurred if the osteotomies were performed after 8 years of age. This is in accordance with other studies that reported better results in younger patients (Hofmann et al. 1982, Ferriter and Shapiro 1987, Loder and Johnston 1987, Doyle et al. 1996, Chotigavanichaya et al. 2002).

Treatment of pronounced deformity is controversial and difficult. An osteotomy to correct varus is inadequate to obtain and maintain satisfactory correction in cases of substantial depression of the medial tibial articular surface (Schoenecker et al. 1992). This is supported by our case, where recurrence occurred twice after this operation. When there is extreme sloping of the medial condyle, a transepiphyseal tibial osteotomy with elevation of the medial condyle and bone grafts in the open wedge was described by Langenskiöld and Riska (1964). Other studies have shown relatively good results after similar osteotomies with elevation of the medial tibial plateau (Sasaki et al. 1986, Gregosiewicz et al. 1989, Langenskiöld 1989, Schoenecker et al. 1992). In recent years, elevation osteotomy of the medial tibial condyle has been combined with gradual lengthening using external fixation with the Ilizarov frame or Taylor’s frame (Accadbled et al. 2003, Bar-On et al. 2008, Hefny and Shalaby 2010, Edwards et al. 2017).

The present surgical technique (Støren 1969) differs from that reported by Langenskiöld and Riska (1964). First, the Støren technique, which is indicated only in cases with open medial tibial physis, is intraepiphyseal whereas Langenskiöld used a transepiphyseal technique. Second, solid bone grafts were taken from the iliac crest whereas Langenskiöld used bone grafts from the tibial diaphysis. Third, Støren did not combine the elevation osteotomy with additional procedures, whereas Langenskiöld performed lateral epiphysiodesis of the proximal tibia and fibula. An intraepiphyseal osteotomy similar to Støren’s technique was described by Siffert (1982), although he combined it with a high inverted “V” tibial osteotomy to correct “the existing varus”.

The long-term outcome of our patient was good, with almost normal function and range of knee motion and moderate residual varus after follow-up of 65 years. The case reported by Siffert (1982) had a good outcome at 13 years’ follow-up. Intraepiphyseal elevation osteotomy should be reserved for older patients in whom tibial osteotomy has failed to prevent progressive deformity. Care should be taken to avoid disturbance of bone nutrition with resulting aseptic necrosis of the elevated fragment and to avoid a lesion of the adjacent tibial epiphyseal line with ensuing inhibition of growth (Støren 1969).

A moderate LLD of 1.5 cm, with the affected leg shorter, was seen in our case. After unilateral transepiphyseal osteotomy, where premature closure of the physis occurs, LLD ranging from 1.5 cm to 6.8 cm was seen in 5 of 7 patients (Schoenecker et al. 1992). This indicates that the intraepiphyseal technique with preservation of the physis is preferable in cases with open physis.

Severe varus deformity should be surgically corrected; if left untreated, OA predictably occurs early in life (Hofmann et al. 1982). It is, however, difficult to evaluate the association between deformity following Blount’s disease and OA, because follow-up in most studies is too short. The longest previous follow-up after elevation osteotomy of the tibial plateau seems to be in a patient aged 41 years (Langenskiöld 1989). The follow-up time of our patient was 65 years (patient age 78 years) and thus more than long enough for a proper evaluation of OA. Surprisingly, only moderate OA had developed during this long time. This shows that a good result at skeletal maturity in a patient with severe preoperative varus (Støren 1969) can remain good even with long follow-up if the deformity has been adequately corrected.

In summary, intraepiphyseal osteotomy with elevation of the medial tibial joint surface and bone grafts to the open wedge gave a very good outcome after a follow-up of 65 years. If accurate preoperative planning and surgical technique are carried out, the method can be recommended in older children with pronounced varus deformity.

The authors would also like to thank Øystein H. Horgmo, Oslo University Hospital, for help with the photos in Figure 2.
